# Supplementary material for: Transcriptomic Analyses and Experimental Validation Identified Immune-Related lncRNA–mRNA Pair MIR210HG–BPIFC Regulating the Progression of Hypertrophic Cardiomyopathy
Source: Int J Mol Sci. 2024 Feb 29;25(5):2816. doi: 10.3390/ijms25052816 (PMC10932045; doi:10.3390/ijms25052816)
Supplement: Supplementary file 1 [file ijms-25-02816-s001.zip › FIG.S3.pdf]

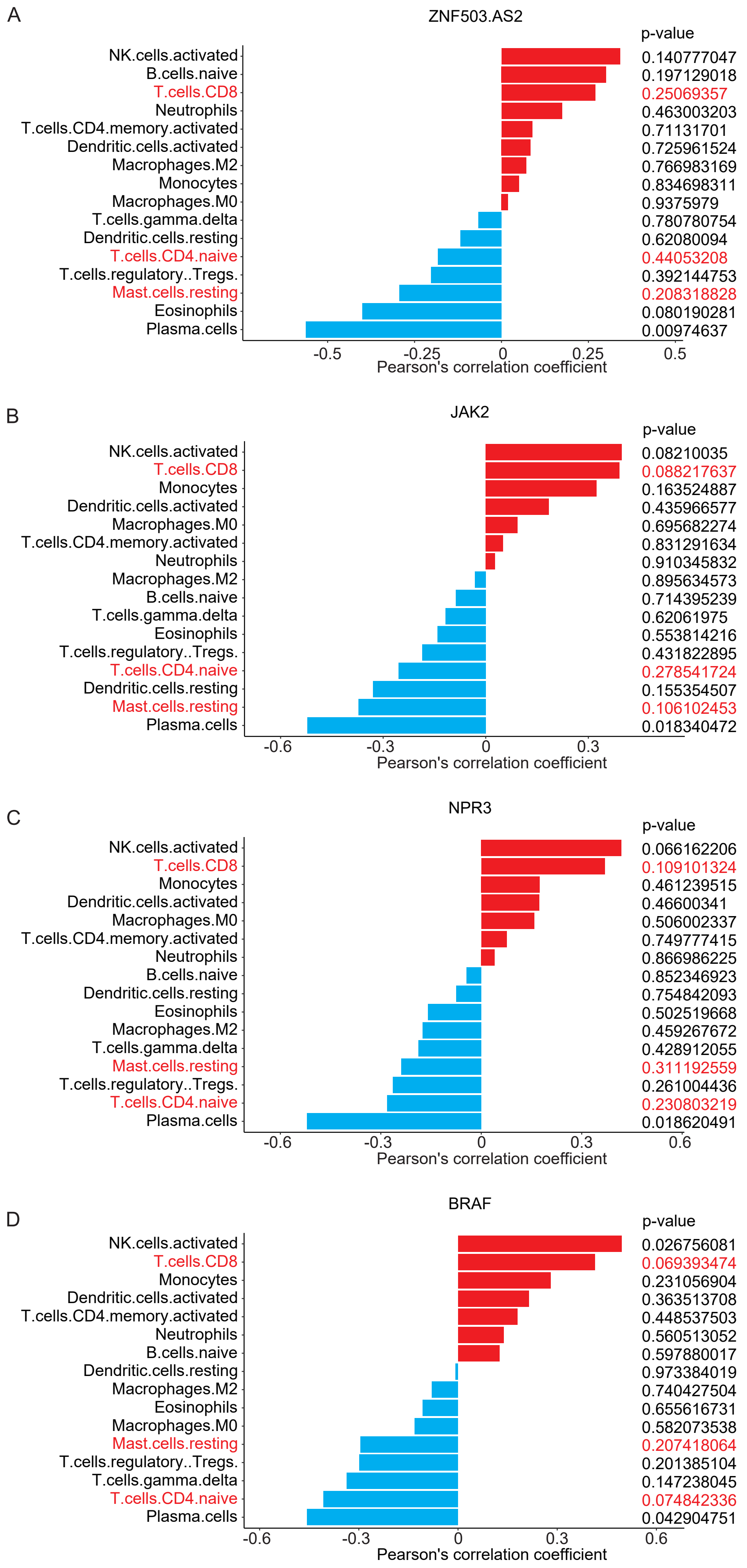

Figure S3 Pearson's correlation analysis of *ZNF503.AS2*, *JAK2*, *NPR3*, *BRAF* and the infiltrating immune cells.
