# Supplementary figures and images for: Transcriptomic Analyses and Experimental Validation Identified Immune-Related lncRNA–mRNA Pair MIR210HG–BPIFC Regulating the Progression of Hypertrophic Cardiomyopathy
Source: Int J Mol Sci. 2024 Feb 29;25(5):2816. doi: 10.3390/ijms25052816 (PMC10932045; doi:10.3390/ijms25052816)

## Sample clustering to GSE180313

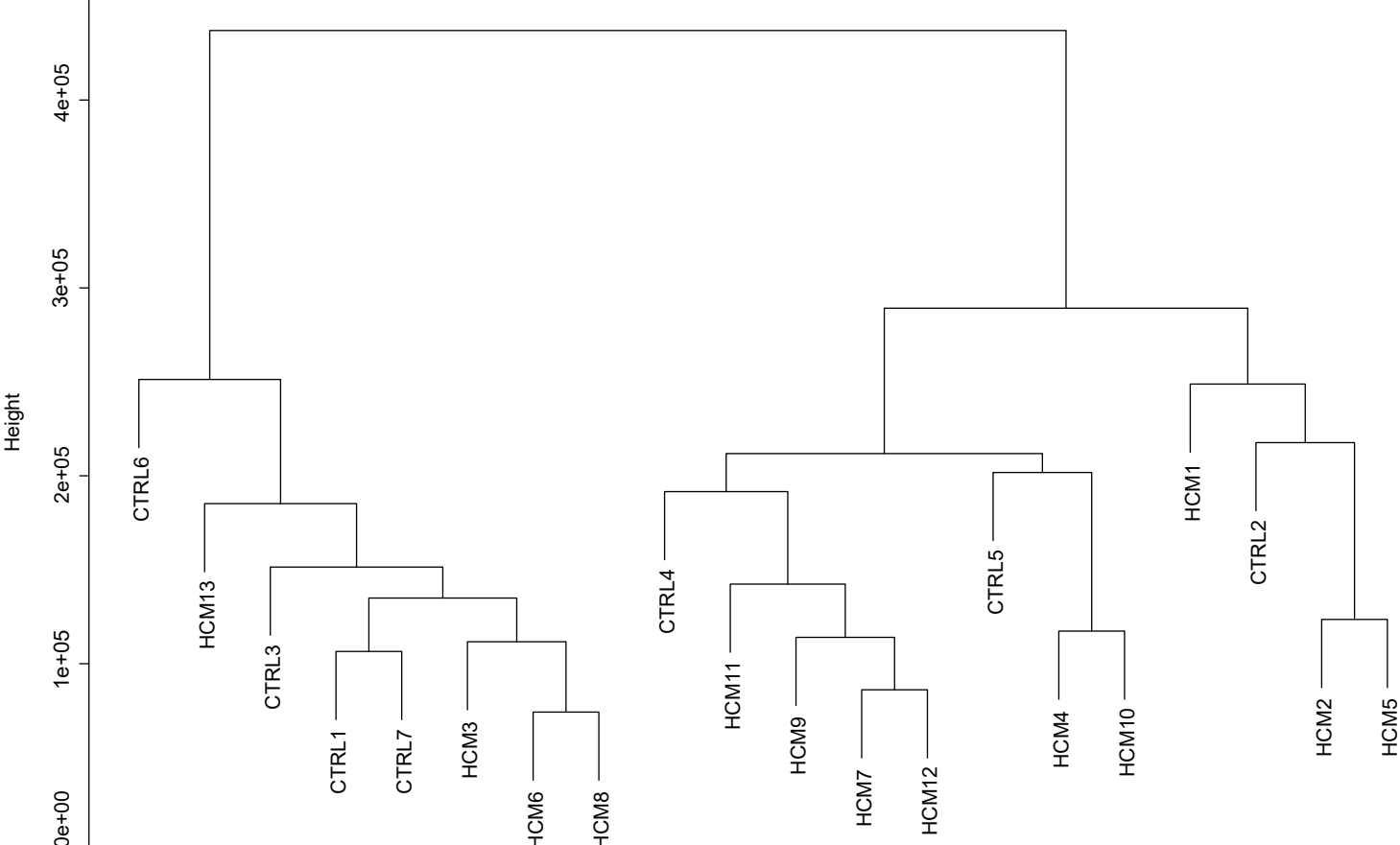

Figure S1 Cluster tree analysis of GSE180313.

Supplement: Supplementary file 1 [file ijms-25-02816-s001.zip › FIG.S1.pdf]

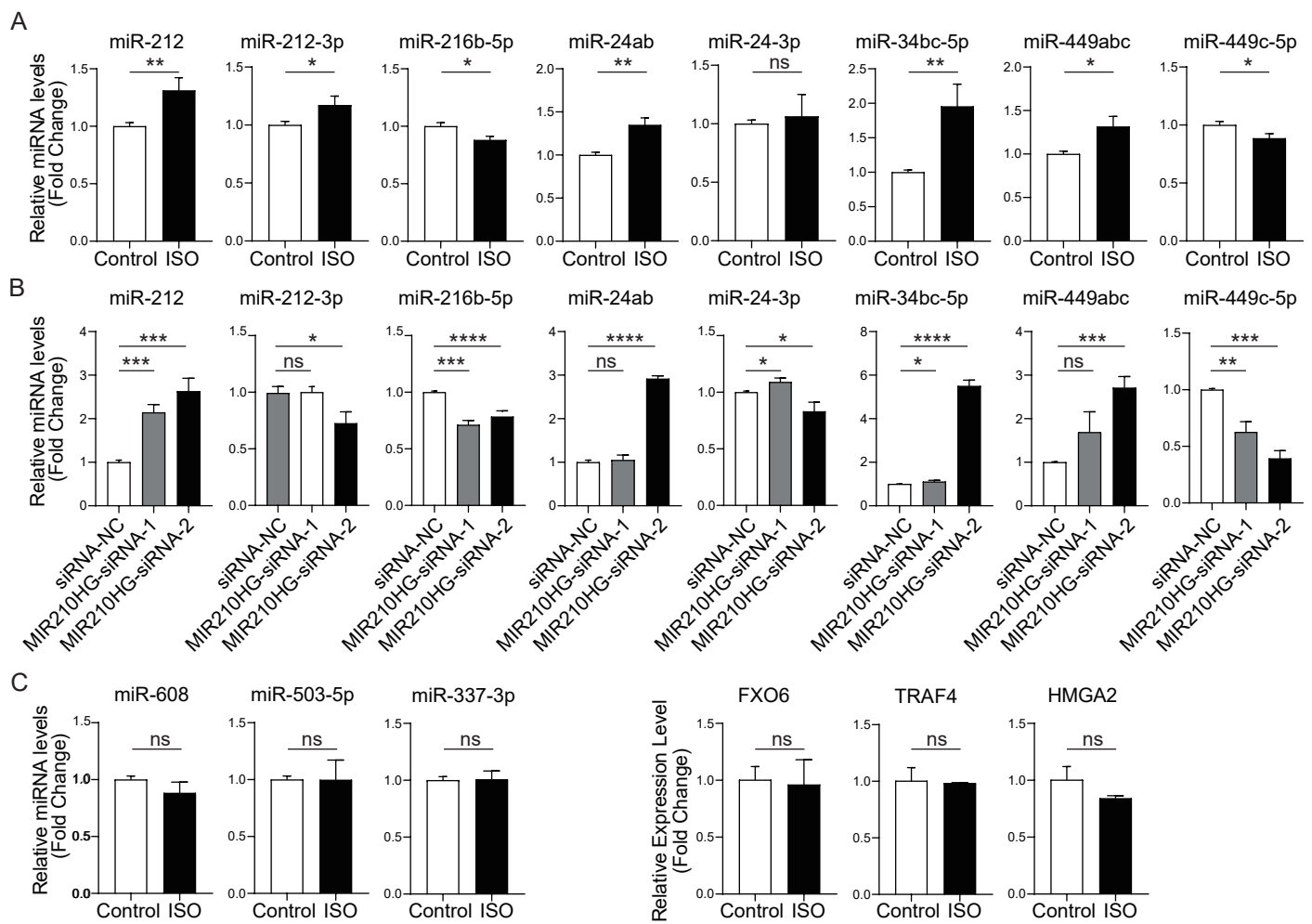

Figure S2 The mRNA and miRNA expression levels.

Supplement: Supplementary file 1 [file ijms-25-02816-s001.zip › FIG.S2-20240219.pdf]
